# The complete nucleotide sequence of chloroplast genome of *Gentiana apiata* (Gentianaceae), an endemic medicinal herb in China

**DOI:** 10.1080/23802359.2019.1641442

**Published:** 2019-07-16

**Authors:** Cai-Xia Huang, Mi-Li Liu, Heng-Jia Zhang, Lei Chang, Yu-Cai Wang, Ji-Xuan Yan

**Affiliations:** a College of Water Conservancy and Hydropower Engineering, Gansu Agricultural University, Lanzhou, China;; b Key Laboratory of Resource Biology and Biotechnology in Western China, Ministry of Education, College of Life Sciences, Northwest University, Shaanxi, China;; c College of Agronomy, Gansu Agricultural University, Lanzhou, China

**Keywords:** *Gentiana apiata*, chloroplast genome, phylogenetic tree

## Abstract

*Gentiana apiata* N. E. Brown (Gentianaceae) is a perennial herb plant and only grows in Qinba Mountains in China. Here, we first characterized the complete nucleotide sequence of chloroplast (cp) genome of *G. apiata* via Illumina next generation sequencing platform. The complete chloroplast genome of *G*. *apiata* was 144,274 bp in length, comprising of a large single copy (LSC) region of 77,353 bp, a small single copy (SSC) region of 17,009 bp, and two inverted repeat regions (IRs) of 24,956 bp. The cp genome contains 127 genes, including 82 protein-coding genes, 35 tRNA, eight rRNA genes, and two pseudogenes. Phylogenetic analysis based on 18 cp genome sequences showed that *G*. *apiata* closely related to congeneric species.

*Gentiana apiata* N. E. Brown (Gentianaceae) is a perennial medicinal herb that only grows in Qinba Mountains in China. It is commonly known as Qinlinglongdan or Zhulingcao in Chinese medicine, and the whole plant has been used as Chinese folk medicine for the treatment of inappetence, hepatic injury disease, and gynaecopathia disease (Zhou et al. 2004). However, little research on this species has been found. In plant, chloroplast (cp) DNA provided valuable phylogenetic information, owning to its conserved genome structures and comparatively high substitution rates (Wu and Ge 2012). In this study, we characterized the complete nucleotide sequence of chloroplast genome of *G*. *apiata* based on the Illumina next-generation sequencing technology.

Fresh samples of *G*. *apiata* were collected from Qingling Mountains in China (N34.0054°, E107.8123°). The genomic DNA was isolated from samples using the modified CTAB method (Doyle and Doyle 1987). DNA sample and voucher specimen (No. GALZH2015722) of *G*. *apiata* were deposited in the Northwest University Museum (NUM). We conducted the next generation sequencing in Novogene Bioinformatics Technology Co., Ltd (Beijing, China). The high-quality reads were obtained after pair-read sequencing. Then, raw reads trimmed and assembled with the program MITObim v 1.8 (Hahn et al. 2013) using *Anemone trullifolia* (NC_039456) as reference. The genome annotation was performed using the online program Dual Organellar Genome Annotator (DOGMA, Wyman et al. 2004) (http://ogdraw.mpimp-golm.mpg.de/). Eventually, the complete and annotated cp genome sequence of *G*. *apiata* has been submitted to GenBank (Accession number: MN049985). Finally, we drew the circular plastid genome maps using the program OGDRAW (Lohse et al. 2013).

The cp genome of *G*. *apiata* is 144,274 bp size, which consist of a large single copy (LSC) region of 77,353 bp, a small single copy (SSC) region of 17,009 bp and two inverted repeat regions (IRs) of 24,956 bp. In total, this cp genome of *G*. *apiata* contains 127 genes, including 82 protein-coding genes, 35 tRNA genes, eight rRNA genes, and two pseudogenes (*ycf1* and *rps19*). Among all the genes, 11 genes (*atpF*, *ndhA*, *ndhB*, *petB*, *rpl2*, *rpl16*, *rpoC1*, *tRNA*-*Leu*, *tRNA*-*Val*, *tRNA*-*Ile*, *tRNA*-*Ala*) contain a single intron and three genes (*ycf3*, *clpP*, *rps12*) contained two introns. The overall GC content of *G*. *apiata* plastome is 37.8%, while the corresponding values of LSC, SSC, and IR regions are 35.6%, 31.4%, and 43.3%, respectively.

A total of 18 cpDNA sequences were used to infer phylogenetic position of *G*. *apiata*. All cpDNA were aligned using the software MAFFT (Katoh and Standley 2013) with the default parameters. The phylogenetic analysis was conducted using the program MEGA7 (Kumar et al. 2016) with 1000 bootstrap replicates. As showed in the phylogenetic tree (Figure 1), the genus *Gentiana* formed a monophyletic clade with high bootstrap supported value (100%), suggesting *G*. *apiata* was closely related to other 14 species of *Gentiana*.

**Figure 1.**
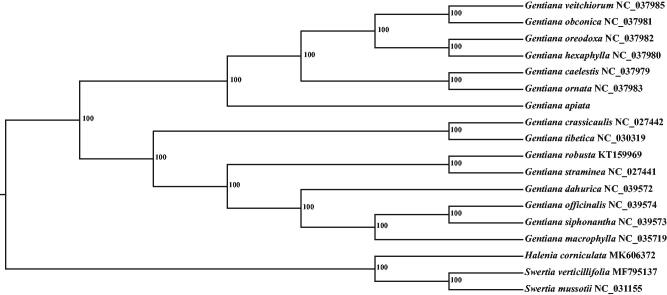
Phylogenetic tree based on 18 complete chloroplast genome sequences.
